# Dorsal Root Ganglion Stimulation Modulates Cortical Gamma Activity in the Cognitive Dimension of Chronic Pain

**DOI:** 10.3390/brainsci10020095

**Published:** 2020-02-11

**Authors:** Tariq Parker, Yongzhi Huang, Ashley L.B. Raghu, James J. FitzGerald, Alexander L. Green, Tipu Z. Aziz

**Affiliations:** Nuffield Department of Surgical Sciences, University of Oxford, Oxford OX3 9DU, UKjames.fitzgerald@nds.ox.ac.uk (J.J.F.); alex.green@nds.ox.ac.uk (A.L.G.); tipu.aziz@nds.ox.ac.uk (T.Z.A.)

**Keywords:** Pain, Dorsal root ganglion stimulation, cognition, gamma, MEG

## Abstract

A cognitive task, the n-back task, was used to interrogate the cognitive dimension of pain in patients with implanted dorsal root ganglion stimulators (DRGS). Magnetoencephalography (MEG) signals from thirteen patients with implanted DRGS were recorded at rest and while performing the n-back task at three increasing working memory loads with DRGS-OFF and the task repeated with DRGS-ON. MEG recordings were pre-processed, then power spectral analysis and source localization were conducted. DRGS resulted in a significant reduction in reported pain scores (mean 23%, *p* = 0.001) and gamma oscillatory activity (*p* = 0.036) during task performance. DRGS-induced pain relief also resulted in a significantly reduced reaction time during high working memory load (*p* = 0.011). A significant increase in average gamma power was observed during task performance compared to the resting state. However, patients who reported exacerbations of pain demonstrated a significantly elevated gamma power (F(3,80) = 65.011612, *p* < 0.001, adjusted *p*-value = 0.01), compared to those who reported pain relief during the task. Our findings demonstrate that gamma oscillatory activity is differentially modulated by cognitive load in the presence of pain, and this activity is predominantly localized to the prefrontal and anterior cingulate cortices in a chronic pain cohort.

## 1. Introduction

Pain is a multi-dimensional experience, traditionally described as consisting of sensory, affective and cognitive domains [1]. Each domain can contribute to the modulation, and at times the propagation, of chronic pain. The cognitive dimension of pain has been demonstrated by investigating the roles that attention, distraction and memory play in altering pain perception [2,3]. Studies have shown that engaging attentional networks with cognitive loads can attenuate perceived pain for a given stimulus — a distraction mechanism of pain relief [4,5]. Conversely, it has also been demonstrated that pain can have a detrimental effect on attentional task performance — a disruptive effect of pain on cognition [6,7]; suggestive of an integrated network involving prefrontal, somatosensory and limbic cortices, and a complex interplay between pain and cognition among these regions.

The role of neurophysiology in these processes has revealed a similarly overlapping feature of pain and cognition—cortical gamma oscillations. High-frequency gamma activity has long been associated with cognition and attention [8,9] but has also been shown to encode ongoing pain [10,11]. Moreover, surgically implanted devices such as spinal cord stimulation have shown the potential to modulate cortical gamma (30–45 Hz) activity [12], supporting the hypothesis of supraspinal mechanisms of action for spinal, and potentially peripheral, neuromodulation.

A key structure of the peripheral nervous system, the dorsal root ganglion (DRG), contains a collection of primary afferent cell bodies in the lateral epidural space which synapse within the spinal cord laminae to convey nociceptive inputs which form the ascending spinothalamic tract. Dorsal root ganglion stimulation (DRGS) is a technique that has gained popularity over the past decade as an effective target of neuromodulation in chronic neuropathic pain and has demonstrated the potential to improve the cognitive-affective dimensions of pain [13]. Neuroimaging has been an invaluable tool to corroborate the effects of cognitive modulation in pain research [14,15,16,17]. As such, we have employed the technique of magnetoencephalography (MEG), coupled with a well-validated working memory task, the *n-back task* [18,19], to investigate the effect of DRGS-mediated pain relief on cognitive performance, the effect of increasing attentional load on the pain percept and the neurophysiologic representation of gamma-band oscillations in a cohort of chronic pain patients.

## 2. Materials and Methods

### 2.1. Participants

The study was conducted with approval from the South-Central Oxford Research Ethics Committee (REF. 13SC0298) in accordance with the Declaration of Helsinki. Sixteen patients were recruited for the study who had undergone surgical implantation of DRG stimulators at the John Radcliffe Hospital for medically refractory chronic pain syndromes (see Table 1). Informed consent was obtained, and participants were randomized, by flipping a coin, to begin MEG recordings in the *ON*-stimulation or *OFF*-stimulation condition, to counter order effects.

### 2.2. Surgical Procedure

**Figure 1:**
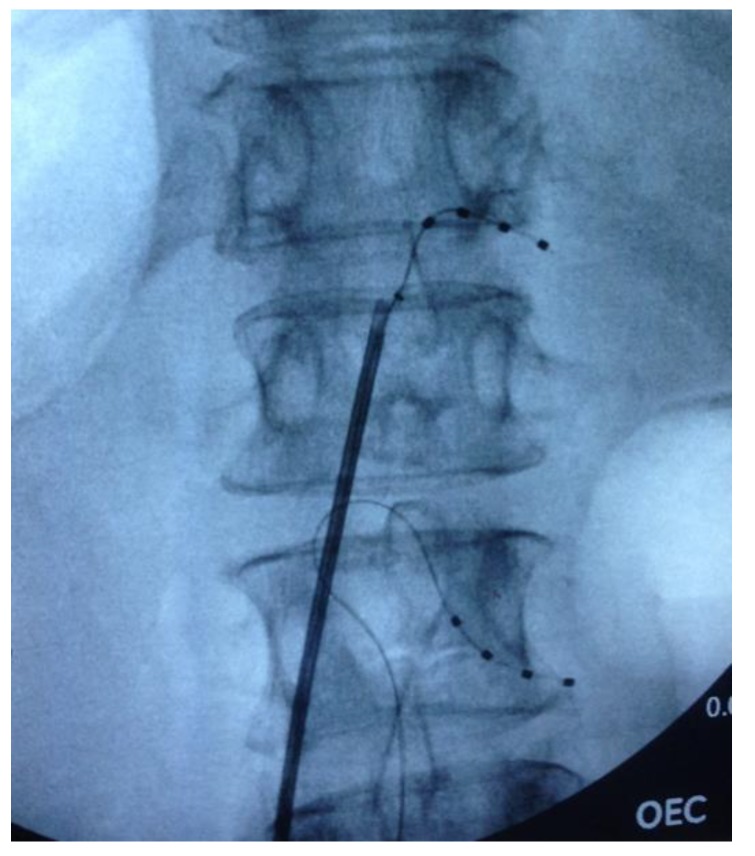
Fluoroscopic image of intra-operative dorsal root ganglion (DRG) lead placement at T12 and L2 on the right side.

The DRG stimulators were implanted under local anaesthetic with light sedation (propofol) in the prone position. Under fluoroscopic control, a delivery sheath was used to enter the epidural space, and a DRG Axium^®^ lead (Abbott Laboratories, Sunnyvale, CA, USA) was introduced under X-ray guidance into the appropriate nerve root exit foramen, so that the electrode contacts were positioned over the dorsum of the DRG in the dorsal part of the foramen. Sedation was weaned and the leads were tested for efficacy prior to re-sedation. Subsequently, when anteroposterior and lateral X-rays had confirmed satisfactory position (See Figure 1), a strain-relief loop was fashioned in the spinal canal, and the wires were tunnelled to an implantable pulse generator (IPG) that was placed subcutaneously remote from the spine.

### 2.3. Attentional Task

A numerical n-back task was used, which consisted of integers ranging from one to four, flashing on a display for 500 msec. Participants were instructed that three working memory loads of increasing difficulty would be cycled for the duration of the task: 0-back, 1-back and 2-back conditions. During the 0-back (low working memory) condition, participants were to immediately respond with a button press corresponding to the number flashed on screen. During the 1-back condition (low-to-intermediate working memory), participants were only to button press if the number flashing on screen corresponded to the number that flashed previously (one back). In the 2-back condition (high working memory), participants were only to button press if the number that flashed on-screen corresponded to the number that appeared two sequences before (two back).

**Figure 2:**
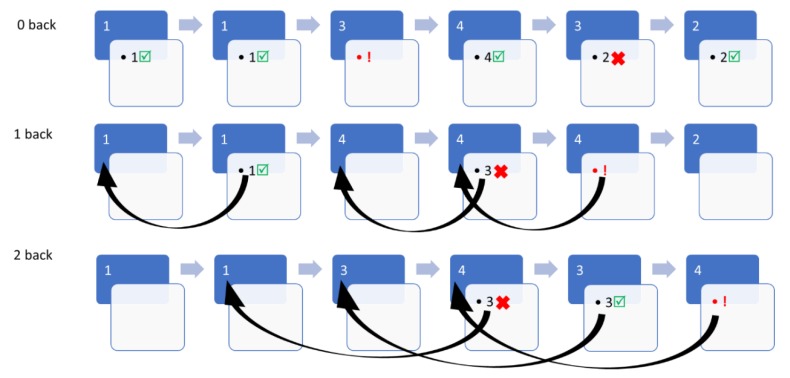
Diagrammatic illustration of numerical n-back task, depicting hits (☑), errors of omission (!) and errors of commission(X) at three working memory loads (0-back, 1-back and 2-back).

Six trials of each condition would cycle sequentially for a total duration of twelve minutes while MEG signals were recorded. Participants were trained until they were comfortable with the paradigm and randomized to start the task in the ON or OFF stimulation condition. The possible outcomes of the task would be a “hit” (correctly identifying a target for the relevant task condition), an error of omission (failure to identify a target for the relevant task condition), an error of commission (incorrectly identifying a non-target as a target in the relevant condition) or no button press (correctly omitting a non-target) (See Figure 2).

Average reaction time (RT) and accuracy (number of hits/total number of targets) for each condition were calculated and evaluated for statistical differences.

### 2.4. Magnetoencephalography

Recordings were performed at the Oxford Centre for Human Brain Activity (OHBA) using a 306-channel Elekta Neuromag MEG system comprised of 102 magnetometers and 204 planar gradiometers at a sampling rate of 1000 Hz. The patient was relaxed and seated under the device, and the relative head position was determined and tracked using Standard Elekta-Neuromag head position indicator (HPI) during the scan. Prior to data acquisition, the HPI coil locations, the position of three anatomical landmarks (the nasion, and left and right pre-auricular points), and the head shape were measured using a three-dimensional digitizer (Polhemus Isotrack). Patients were scanned during the n-back task for 12 min in both DRGS-ON and DRGS-OFF conditions, separated by a pre-defined washout period [20] to prevent carryover effects. Patients were also scanned with the DRGS-OFF at rest with eyes open for comparison with task conditions. Electrocardiographic (ECG) recordings were monitored by applying bilateral electrodes to the volar aspect of the wrists and, simultaneously, electrooculographic (EOG) traces were recorded by two electrodes, placed above and below the left eye.

### 2.5. Spectral and Source Analysis

Data were visually inspected and artefacts such as flats and jumps were detected in each channel and marked. The strong magnetic artefacts in the raw data, such as the artefacts of stimulation, were suppressed by the spatiotemporal signal space separation (tSSS) method [21] with a subspace correlation limit of 0.9 [22,23] using MaxFilter software (Elekta Neuromag, version 2.2). Additionally, the automatic detection of saturated and bad MEG channels was also applied in the software. The bad channels detected were excluded from tSSS analysis to prevent artefacts spreading. The resultant MEG data were analysed with MATLAB R2019a using the Fieldtrip [24] and Brainstorm [25] toolboxes. The raw MEG data was filtered between 1–100 Hz and a bandstop filter of 48–52 Hz was also applied before recordings were resampled to 300 Hz. Independent Component Analysis (ICA) was used to decompose the MEG data, identify and subsequently remove eye-blink and cardiac artefacts. The components related to eye-blink and cardiac activity were identified by comparing the ICA component with the EOG and ECG recordings.

The power spectra were estimated using Welch’s method with a Hanning window of 3 s with a 50% overlap. The relevant epochs were then extracted for each working memory load condition and power spectral density (PSD) estimates averaged across all MEG channels. PSDs were then normalized by dividing by the integral power between 1 Hz and 50 Hz to control for inherent differences within each participant and the average power spectra binned to the frequency of interest-gamma band activity (30–45Hz).

The implanted DRG stimulators used were not MRI compatible and, as such, individual structural MRIs (pre- or post-operative) were not available. Therefore, the ICB152 MRI template in Brainstorm was warped to fit the head model of each participant by co-registering the nasion, left and right pre-auricular fixed points acquired during head shape digitization [26]. Each subject-specific template was then used to calculate a lead field matrix based on a single shell model. The subsequent head model was co-registered with MEG data, and source localization performed using the dynamical imaging of coherent sources (DICS) beamformer technique based on the frequency of interest (30–45 Hz) of the processed MEG signals.

### 2.6. Statistical Analysis

Statistical analyses of MEG data to determine normalized PSD differences between *ON* and *OFF* stimulation was based on the non-parametric cluster-based permutation tests in the Fieldtrip toolbox [27]. A cluster was defined as two or more adjacent sensors reaching the pre-determined level of significance (*t*-statistic < 0.05). Statistical significance determined using the Monte Carlo method (*p*-value < 0.05, two-tailed) in order to correct for multiple comparisons. Comparisons of relative power between resting state and task performance conditions were calculated by finding the difference in the relative power between the two conditions and normalizing to the baseline power of the resting state condition to correct for inter-subject variability. The GraphPad Prism software version 8.1 (La Jolla California, CA, USA, www.graphpad.com) was used for other figures and statistical analyses presented. D’Agostino normality testing was conducted on each data set to confirm Gaussian distribution and the corresponding parametric test — Student’s *t*-test or mixed-effects ANOVA (for comparisons of three or more groups) were utilized for analyses, respectively. *P*-values < 0.05 were regarded as statistically significant.

### 2.7. Mediation Analysis

A two-tailed Pearson correlation was performed to identify the relationship between gamma-band activity and patients’ reported pain scores and task reaction times. Mediation analysis was conducted using SPSS (version 26) to assess whether there was a mediating effect between pain-related and cognition-related gamma activity in the frontal cortex, somatosensory cortex and dorsolateral prefrontal cortex. Mediation was tested by means of the joint significance test [28].

## 3. Results

**Figure 3:**
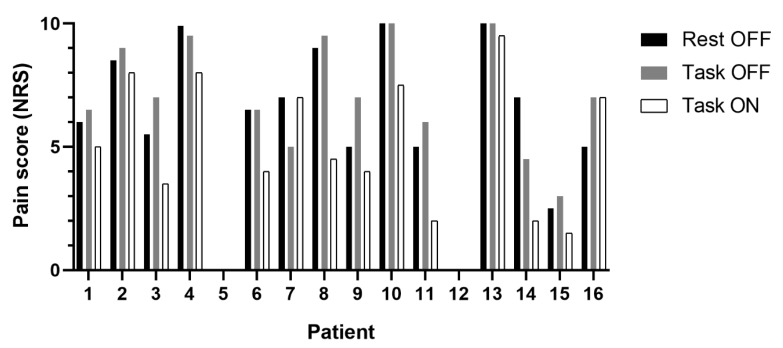
Grouped column graph depicting change from baseline pain scores at rest (black) and during n-back task performance (grey) with dorsal root ganglion stimulators (DRGS) turned off, as well as during task performance with DRGS turned on (white) among the sixteen participants. Of note, patients 5 and 12 had mobility-associated/posture-dependent pain and served as a unique “no-pain control” for the study.

Sixteen participants were recruited (10 males, 6 females) with an average age of 51 years (SD 16.5). However, only thirteen patients were included in MEG analysis after excluding data with unacceptable artefact/missing MEG channels. Contrary to expectation, only three of the sixteen participants reported alleviation of pain during task performance during the *DRGS-OFF* condition. The majority reported either worsening of pain scores (*n* = 8), or no change in pain (*n* = 3) during task performance compared to rest (see Figure 3). Interestingly, our cohort also included patients with posture-dependent/mobility-associated chronic pain syndromes (*n* = 2), which meant they did not report any pain at rest or during the task performance.

However, there was a significant reduction in reported pain scores (mean reduction 23% (SD 0.27), (F(2,30) = 10.33, *p* = 0.001) when DRGS was switched *ON* during the task, compared to *DRGS-OFF* during rest (*p* = 0.01) and task conditions (*p* = 0.005) (See Figure 3).

### 3.1. Task Performance

**Figure 4:**
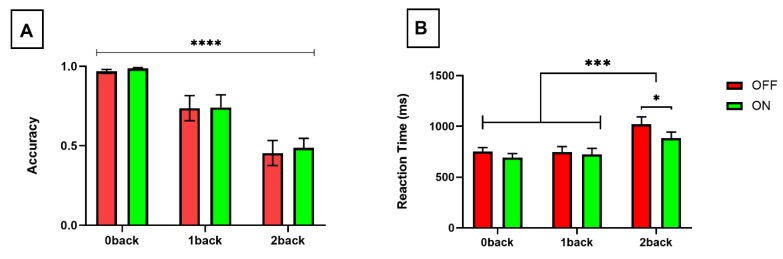
Bar graphs illustrating (**A**) task accuracy (proportion of correctly identified hits of all targets presented) and (**B**) reaction time with DRGS OFF (red) and ON (green) over increasing working memory loads. *p* < 0.0001 - ****; *p* < 0.001 - ***; *p* < 0.05 - *.

There was a significant reduction in task accuracy (F(2,24) = 36.25, *p* < 0.0001) (See Figure 4A) and prolongation of RT (F(2,24) = 14.59, *p* < 0.0001) (See Figure 4B) in response to increasing attentional loads. There was no significant difference in RTs between 0-back and 1-back conditions, regardless of stimulation condition (OFF stimulation, *p* = 0.98, ON stimulation *p* = 0.73). However, the effect of working memory load on RT was driven by differences between the two lower working memory loads (0-back/1-back) and high working memory load (2-back) for both OFF (*p* < 0.001) and ON (*p* = 0.004) stimulation conditions (See Figure 4B).

DRG stimulation was associated with a significant reduction in reaction time (F(1,12) = 6.516, *p* = 0.025), with posthoc tests confirming the statistical difference within the highest working memory load (2-back) condition (*p* = 0.011) (See Figure 4B). In contrast, there was no significant difference in task accuracy in response to DRGS across any working memory load condition (F(1,12) = 0.722, *p* = 0.41) (See Figure 4A).

### 3.2. Gamma Band Activity

**Figure 5:**
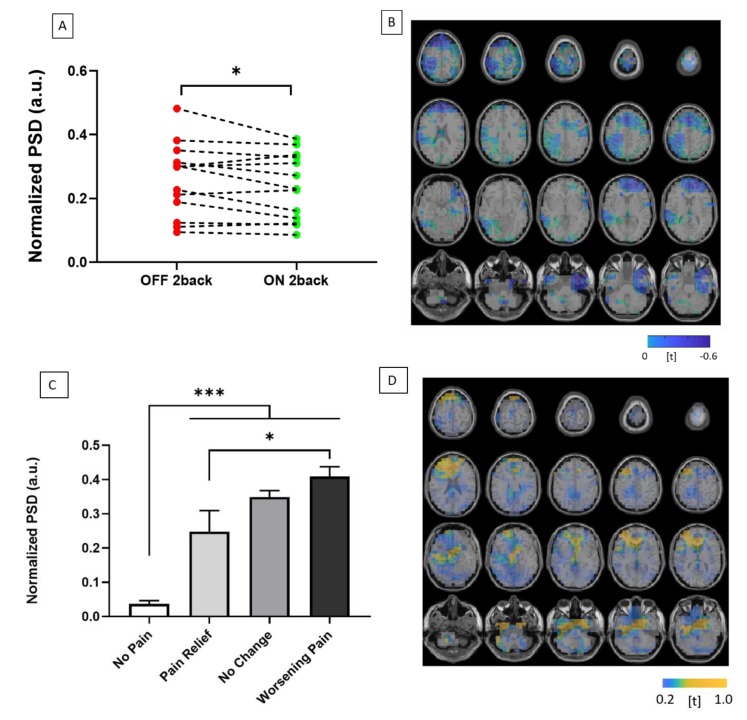
(**A**) Graph illustrating change in normalized power spectral density (PSD) between OFF (red) and ON (green) DRGS during high cognitive load (2-back condition). (**B**) 3-D source localization demonstrating t-statistic maps of significant reductions in gamma cortical activity across the prefrontal, anterior cingulate and somatosensory cortices during DRGS-mediated pain relief. (**C**) Column graph illustrating change in normalized power spectral density (PSD) with DRGS OFF, during high working memory load (2-back condition) compared to resting-state, grouped according to pain response during working memory load: no pain (*n* = 2), pain relief (n = 2), no change (*n* = 3) and worsening pain (*n* = 6) groups (A total of 13 patients were included in the MEG analysis). (**D**) 3-D source localization demonstrating t-maps, as before, of significant increases in cortical activity across the prefrontal and anterior cingulate cortices during task performance.

Of the patients included in the MEG analysis experiencing pain during the study (*n* = 11), five reported 50% or greater reduction in reported pain scores with DRGS, while one reported worsening of pain. DRGS-mediated pain relief was associated with a significant reduction in gamma activity (30–45 Hz) across all MEG sensors during task performance (*t* = 2.27, *p* = 0.036) (See Figure 5A). The observed reduction in gamma band activity during pain relief was predominantly localized to the prefrontal cortex based on source-space analyses, but also revealed reductions in gamma activity in both somatosensory and anterior cingulate cortices after 3D source reconstruction (See Figure 5B).

**Figure 6:**
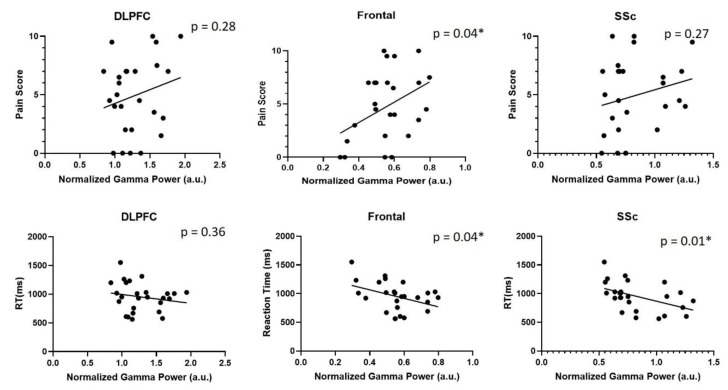
Graphs depicting correlations between reported pain scores and normalized gamma activity (top row), as well as correlations between reaction time and 2-back reaction times (bottom row) in the dorsolateral prefrontal cortex, frontal cortex and somatosensory cortex (SSc).

There were significant differences in gamma band fluctuations, dependent on the interaction of distraction and pain scores (F(3,80) = 65.01, *p* < 0.001). All groups exhibited increased gamma oscillatory activity during task performance compared to resting state. There was significantly greater gamma activity during task performance among those patients experiencing pain compared to pain-free controls (*p* < 0.001) (see Figure 6). Furthermore, among those in the pain-state, there was a significantly greater change in gamma oscillatory activity in patients that reported worsening pain during the task compared to those that exhibited pain relief during the attentional task (*p* = 0.01) (See Figure 5C). This increased gamma activity was also localized to the prefrontal and anterior cingulate cortices (See Figure 5D).

A significant correlation was found between gamma-band activity and subjectively reported pain scores in the frontal cortex([*r* = 0.4, *p* = 0.04). Additionally, significant correlations were observed between gamma-band activity and reaction times in both frontal and somatosensory cortices (See Figure 6). However, further analysis did not reveal a mediating effect of pain on cognition, or vice-versa (See Table 2).

## 4. Discussion

Our findings demonstrate the efficacy of DRGS in alleviating the interruptive effect of pain on cognition and supports the use of neurophysiologic signals, in particular, gamma-band activity, to interrogate the cognitive dimension of pain. We further demonstrate that while increased cognitive load is reflected by enhanced gamma oscillatory activity, the effect of pain, and pain relief, can modulate gamma activity in the human prefrontal and anterior cingulate cortices. Furthermore, our findings demonstrate that while frontal gamma activity was correlated with pain and cognitive measures, there was no mediating effect of pain on cognition, or vice-versa, which suggests that the potential for pain and cognition to modulate cortical gamma activity occur independently.

An inverse relationship is to be expected between task accuracy and reaction time with increasing cognitive load [29]. Accordingly, the n-back task results in our chronic pain cohort showed a significant reduction in task accuracy and a concomitant increase in reaction times with increasing working memory loads. However, cognitive loading (working memory) failed to alleviate pain in the majority of our participants. The phenomenon of distraction-induced analgesia is equivocal, having demonstrated mixed results across the pain literature. While there are studies which suggest that selective attention can mitigate the sensation of pain [14,30], there are also studies which have found that distraction can also exacerbate the perception of pain [31], as was seen in seven of the sixteen participants recruited in this study. Interestingly, the studies which demonstrate the phenomenon of distraction-mediated analgesia have been performed in healthy adults with the application of experimentally-induced pain. However, the initial report of worsened post-distraction pain [31], was performed in a cohort of chronic back pain patients which, taken together with our findings, suggests that this mechanism of pain alleviation may not be as applicable in chronic pain as previously thought.

It is classically believed that attention processing has a limited capacity, and by re-directing a portion of attentional reserves towards a cognitively demanding exercise, such as the n-back task, the accessibility of pain processing to this attentional network is decreased [32,33,34]. However, this mechanism of attentional switching seems to be sensitive to the degree of pain and the demands of the task on central attention [35,36]. A pleasant, moderately-engaging task might produce the intended alleviation of the pain percept by gating the accessibility of salient noxious stimuli to conscious processing. However, it seems similarly plausible that the challenge of a difficult, cognitively-demanding task can become frustrating and potentially exacerbate pain perception.

The disruptive effect of pain on task performance (accuracy) was not found to be significant in our cohort, despite marginal increases in accuracy during therapeutic DRGS. However, participants’ reaction times were significantly reduced for a given level of accuracy, particularly in the high working memory load (2-back) condition. This suggests that with the alleviation of chronic pain, reduced response latency can be achieved without sacrificing task performance. Pain is a well-known interruptive factor in cognitive performance [37,38,39], and, persons suffering from chronic pain have been shown to exhibit deficits in various aspects of cognitive function including attention and memory [40,41]. The impact of pain on cognition seems to be dependent on the attentional load required of the task [42,43], which has similarly been demonstrated by our findings. The majority of these studies have been conducted with experimentally-induced pain in healthy adult participants. However, our study benefited from the ability to investigate the effect of acute pain relief, through neuromodulation, within the chronic pain phenotype and demonstrated its ability to improve performance on a cognitive task.

Our findings are bolstered by incorporating a well-established neurophysiologic signature, gamma oscillatory activity, as an objective metric of pain and attention. The neurophysiologic importance of gamma-band activity in the attentional modulation of pain has been previously demonstrated in healthy controls [44]. While our findings support the academic consensus which describes increased gamma activity in response to increased attentional demands [45,46], we further delineate the potential for pain to modulate this gamma activity.

DRGS-induced pain relief was associated with significantly reduced gamma activity during task performance (See Figure 5A). While a previous MEG study of SCS has hypothesized about the potential for increased cortical gamma activity in chronic pain [47], our findings have provided further support for this proposed mechanism of thalamocortical dysrhythmia. Our study also benefitted from a “no-pain control” group in this chronic pain cohort. Interestingly, in the *DRGS-OFF* condition, the “no-change” and “no-pain” groups also showed a significant disparity in gamma activity despite neither group having reported benefit from distraction-mediated analgesia (See Figure 5C). This observation suggests that this increased gamma activity is representative of ongoing pain in the chronic pain cortical network of the “no-change” group. Furthermore, we observed significantly lower gamma activity among participants reporting pain relief during task performance, compared to those reporting worsening pain (See Figure 5C). Taken altogether, our results suggest that the blunted increase in gamma activity we observed during task performance is likely a consequence of pain alleviation from distraction. However, it is also possible that pain relief in this group occurred in response to distraction-mediated analgesia, and this dampened gamma activity may represent the diversion of limited attentional resources. Further studies are required to conclusively disambiguate the causal relationship between these two possibilities.

The results of MEG source localization revealed gamma activation in brain regions which are known to be involved in the overlapping network of pain and attention, including somatosensory cortex [48,49] and cingulate cortices [50,51]. However, the observed changes in gamma activity were predominantly localized to the prefrontal cortex, which has been implicated in the top-down attentional modulation of painful stimuli [52] and has also been identified as a region that encodes ongoing pain among chronic pain patients and healthy adults [53,54]. Similar findings of attenuated cortical activity in cortico-limbic networks during DRG stimulation has been demonstrated in pre-clinical studies [55] and EEG studies of SCS [56]. Coupled with our findings of increased gamma activity during cognitive loads, and decreased gamma activity during pain relief in the prefrontal cortex, this represents further supportive evidence of the supraspinal effects of DRG stimulation.

The authors acknowledge the study limitations of a small sample size, resulting from the novelty of DRGS as an intervention for chronic pain. However, the utilization of a crossover study design was employed to overcome this limitation and increase statistical power by minimizing between-subject variability. We also recognize that such an overlap in cortical networks between pain-related and attention-related activities may still be represented by more functionally distinct anatomical regions than the areas described in our analysis. Further elucidation of these anatomical differences might be achieved by combining techniques such as fMRI which can resolve deeper anatomical structures involved in the pain network (insular cortex, thalamus) with greater sensitivity and spatial resolution. These limitations notwithstanding, this study offers novel evidence for the supraspinal effects of DRGS in chronic pain and demonstrates the importance of gamma oscillatory activity in the neurophysiologic representation of pain and cognition.

## Figures and Tables

**Table 1:** Patient demographics and DRG stimulation parameters, CRPS—Complex regional pain syndrome.

Patient		Age	Gender	Diagnosis	Electrode Location	Stimulation Parameters (Frequency (Hz)/Amplitude (mA)/Pulse Width (μs))
**1**		49	Female	Postherpetic neuralgia	Right L5	20/1.6/400
**2**		53	Female	Meralgia paresthetica	Right L2	20/0.6/300
**3**		29	Male	Post-traumatic compressive neuropathy	Left L2	20/0.7/250
**4**		78	Male	Diabetic neuropathy	Bilateral L5	Right - 20/1.025/450 Left - 20/0.775/480
**5**		46	Male	CRPS	Right L3	20/0.7/410
**6**		52	Male	Post-operative nerve entrapment	Left L1	28/1.3/250
**7**		58	Female	CRPS	Right L2/L3	20/2.1/250
**8**		61	Male	Post-operative mononeuropathy	Left L3	20/2.1/140
**9**		47	Male	CRPS	Left L4	20/6/350
**10**		55	Male	Nerve entrapment	Right C7/C8	20/0.425/300
**11**		29	Male	Post-operative radiculopathy	Bilateral L5	Right - 20/2.25/700, Left - 20/650/800
**12**		52	Female	CRPS	Right L5	30/0.7/500
**13**		77	Female	Postherpetic neuralgia	Right T1	30/0.4/300
**14**		22	Female	Dystonic pain	Right L2/L3	20/2.4/300
**15**		52	Male	Post-operative mononeuropathy	Right L1	30/0.525/400
**16**		54	Male	Post-operative radiculopathy	Right L3/L4	20/0.475/360

**Table 2:** Mediation effects between pain-related gamma activity and cognition-related gamma activity in frontal, somatosensory and dorsolateral prefrontal cortices.

	Standardized β	Standard Error	*p*-value
**Frontal**			
Pain → Gamma	0.398	0.008	0.044
Cognition → Gamma	−0.332	0.00	0.082
**Somatosensoy cortex**			
Pain → Gamma	0.93	0.014	0.63
Cognition → Gamma	−0.447	0.00	0.028
**Dorsolateral Prefrontal cortex**			
Pain → Gamma	0.179	0.019	0.4
Cognition → Gamma	−0.134	0.00	0.53

## References

[1] Melzack R., Casey K., Thomas C.C. (1968). Sensory, Motivational, and Central Control Determinants of Pain. The Skin Senses: Proceedings.

[2] Bushnell M.C., Čeko M., Low L.A. (2013). Cognitive and emotional control of pain and its disruption in chronic pain. Nat. Rev. Neurosci..

[3] Torta D.M., Legrain V., Mouraux A., Valentini E. (2017). Attention to pain! A neurocognitive perspective on attentional modulation of pain in neuroimaging studies. Cortex.

[4] Schreiber K.L., Campbell C., Martel M.O., Greenbaum S., Wasan A.D., Borsook D., Jamison R.N., Edwards R.R. (2014). Distraction analgesia in chronic pain patients the impact of catastrophizing. Anesthesiology.

[5] Valet M., Sprenger T., Boecker H., Willoch F., Rummeny E., Conrad B., Erhard P., Tolle T.R. (2004). Distraction modulates connectivity of the cingulo-frontal cortex and the midbrain during pain—An fMRI analysis. Pain.

[6] Berryman C., Stanton T.R., Jane Bowering K., Tabor A., McFarlane A., Lorimer Moseley G. (2013). Evidence for working memory deficits in chronic pain: A systematic review and meta-analysis. Pain.

[7] Eccleston C., Crombez G. (1999). Pain demands attention: A cognitive-affective model of the interruptive function of pain. Psychol. Bull..

[8] Gruber T., Müller M.M., Keil A., Elbert T. (1999). Selective visual-spatial attention alters induced gamma band responses in the human EEG. Clin. Neurophysiol..

[9] Tallon-Baudry C., Bertrand O., Hénaff M.-A., Isnard J., Fischer C. (2005). Attention Modulates Gamma-band Oscillations Differently in the Human Lateral Occipital Cortex and Fusiform Gyrus. Cereb. Cortex.

[10] De Pascalis V., Cacace I., Massicolle F. (2004). Perception and modulation of pain in waking and hypnosis: Functional significance of phase-ordered gamma oscillations. Pain.

[11] Tan L.L., Oswald M.J., Heinl C., Retana Romero O.A., Kaushalya S.K., Monyer H., Kuner R. (2019). Gamma oscillations in somatosensory cortex recruit prefrontal and descending serotonergic pathways in aversion and nociception. Nat. Commun..

[12] Bai Y., Xia X., Liang Z., Wang Y., Yang Y., He J., Li X. (2017). Frontal Connectivity in EEG Gamma (30–45 Hz) Respond to Spinal Cord Stimulation in Minimally Conscious State Patients. Front. Cell. Neurosci..

[13] Deer T.R., Levy R.M., Kramer J., Poree L., Amirdelfan K., Grigsby E., Staats P., Burton A.W., Burgher A.H., Obray J. (2017). Dorsal root ganglion stimulation yielded higher treatment success rate for complex regional pain syndrome and causalgia at 3 and 12 months: A randomized comparative trial. Pain.

[14] Bantick S.J., Wise R.G., Ploghaus A., Clare S., Smith S.M., Tracey I. (2002). Imaging how attention modulates pain in humans using functional MRI. Brain.

[15] Tracey I., Ploghaus A., Gati J.S., Clare S., Smith S., Menon R.S., Matthews P.M. (2002). Imaging Attentional Modulation of Pain in the Periaqueductal Gray in Humans. J. Neurosci..

[16] Seminowicz D.A., Davis K.D. (2007). Interactions of pain intensity and cognitive load: The brain stays on task. Cereb. Cortex.

[17] Sprenger C., Eippert F., Finsterbusch J., Bingel U., Rose M., Büchel C. (2012). Attention Modulates Spinal Cord Responses to Pain. Curr. Biol..

[18] Moore D.J., Keogh E., Eccleston C. (2013). The effect of threat on attentional interruption by pain. Pain.

[19] Attridge N., Noonan D., Eccleston C., Keogh E. (2015). The disruptive effects of pain on n-back task performance in a large general population sample. Pain.

[20] Parker T., Green A., Aziz T. (2019). Rapid onset and short washout periods of dorsal root ganglion stimulation facilitate multiphase crossover study designs. Brain Stimul..

[21] Taulu S., Simola J. (2006). Spatiotemporal signal space separation method for rejecting nearby interference in MEG measurements. Phys. Med. Biol..

[22] Medvedovsky M., Taulu S., Bikmullina R., Ahonen A., Paetau R. (2009). Fine tuning the correlation limit of spatio-temporal signal space separation for magnetoencephalography. J. Neurosci. Methods.

[23] Carrette E., De Tiège X., Op De Beeck M., De Herdt V., Meurs A., Legros B., Raedt R., Deblaere K., Van Roost D., Bourguignon M. (2011). Magnetoencephalography in epilepsy patients carrying a vagus nerve stimulator. Epilepsy Res..

[24] Oostenveld R., Fries P., Maris E., Schoffelen J.M. (2011). FieldTrip: Open source software for advanced analysis of MEG, EEG, and invasive electrophysiological data. Comput. Intell. Neurosci..

[25] Tadel F., Baillet S., Mosher J.C., Pantazis D., Leahy R.M. (2011). Brainstorm: A user-friendly application for MEG/EEG analysis. Comput. Intell. Neurosci..

[26] Gohel B., Lim S., Kim M.-Y., Kwon H., Kim K. (2017). Approximate Subject Specific Pseudo MRI from an Available MRI Dataset for MEG Source Imaging. Front. Neuroinform..

[27] Maris E., Oostenveld R. (2007). Nonparametric statistical testing of EEG- and MEG-data. J. Neurosci. Methods.

[28] MacKinnon D.P., Lockwood C.M., Hoffman J.M., West S.G., Sheets V. (2002). A comparison of methods to test mediation and other intervening variable effects. Psychol. Methods.

[29] Meule A. (2017). Reporting and interpreting task performance in Go/no-go affective shifting tasks. Front. Psychol..

[30] Buhle J., Wager T.D. (2010). Performance-dependent inhibition of pain by an executive working memory task. Pain.

[31] Goubert L., Crombez G., Eccleston C., Devulder J. (2004). Distraction from chronic pain during a pain-inducing activity is associated with greater post-activity pain. Pain.

[32] McCaul K.D., Malott J.M. (1984). Distraction and coping with pain. Psychol. Bull..

[33] Kahneman D. (1973). Attention and Effort.

[34] Broadbent D. (1958). Perception and Communication.

[35] Eccleston C. (1995). Chronic pain and distraction: An experimental investigation into the role of sustained and shifting attention in the processing of chronic persistent pain. Behav. Res. Ther..

[36] Roa Romero Y., Straube T., Nitsch A., Miltner W.H.R., Weiss T. (2013). Interaction between stimulus intensity and perceptual load in the attentional control of pain. Pain.

[37] Moore D.J., Keogh E., Eccleston C. (2013). Headache impairs attentional performance. Pain.

[38] Keogh E., Cavill R., Moore D.J., Eccleston C. (2014). The effects of menstrual-related pain on attentional interference. Pain.

[39] Attridge N., Keogh E., Eccleston C. (2016). The effect of pain on task switching: Pain reduces accuracy and increases reaction times across multiple switching paradigms. Pain.

[40] Nadar M.S., Jasem Z., Manee F.S. (2016). The cognitive functions in adults with chronic pain: A comparative study. Pain Res. Manag..

[41] Moriarty O., Ruane N., O’Gorman D., Maharaj C.H., Mitchell C., Sarma K.M., Finn D.P., McGuire B.E. (2017). Cognitive impairment in patients with chronic neuropathic or radicular pain: An interaction of pain and age. Front. Behav. Neurosci..

[42] Moore D.J., Keogh E., Eccleston C. (2012). The Interruptive Effect of Pain on Attention. Q. J. Exp. Psychol..

[43] Moore D.J., Eccleston C., Keogh E. (2017). Cognitive load selectively influences the interruptive effect of pain on attention. Pain.

[44] Hauck M., Lorenz J., Engel A.K. (2007). Attention to painful stimulation enhances γ-band activity and synchronization in human sensorimotor cortex. J. Neurosci..

[45] Jensen O., Kaiser J., Lachaux J.P. (2007). Human gamma-frequency oscillations associated with attention and memory. Trends Neurosci..

[46] Ray S., Niebur E., Hsiao S.S., Sinai A., Crone N.E. (2008). High-frequency gamma activity (80–150 Hz) is increased in human cortex during selective attention. Clin. Neurophysiol..

[47] Schulman J.J., Ramirez R.R., Zonenshayn M., Ribary U., Llinas R. (2005). Thalamocortical dysrhythmia syndrome: MEG imaging of neuropathic pain. Thalamus Relat. Syst..

[48] Petrovic P., Ingvar M., Stone-Elander S., Petersson K.M., Hansson P. (1999). A PET activation study of dynamic mechanical allodynia in patients with mononeuropathy. Pain.

[49] Petrovic P., Petersson K.M., Ghatan P.H., Stone-Elander S., Ingvar M. (2000). Pain-related cerebral activation is altered by a distracting cognitive task. Pain.

[50] Davis K.D., Taylor S.J., Crawley A.P., Wood M.L., Mikulis D.J. (1997). Functional MRI of pain- and attention-related activations in the human cingulate cortex. J. Neurophysiol..

[51] Schmidt-Wilcke T., Kairys A., Ichesco E., Fernandez-Sanchez M.L., Barjola P., Heitzeg M., Harris R.E., Clauw D.J., Glass J., Williams D.A. (2014). Changes in clinical pain in fibromyalgia patients correlate with changes in brain activation in the cingulate cortex in a response inhibition task. Pain Med. (USA).

[52] Legrain V., Van Damme S., Eccleston C., Davis K.D., Seminowicz D.A., Crombez G. (2009). A neurocognitive model of attention to pain: Behavioral and neuroimaging evidence. Pain.

[53] Schulz E., May E.S., Postorino M., Tiemann L., Nickel M.M., Witkovsky V., Schmidt P., Gross J., Ploner M. (2015). Prefrontal Gamma Oscillations Encode Tonic Pain in Humans. Cereb. Cortex.

[54] May E.S., Nickel M.M., Ta Dinh S., Tiemann L., Heitmann H., Voth I., Tölle T.R., Gross J., Ploner M. (2019). Prefrontal gamma oscillations reflect ongoing pain intensity in chronic back pain patients. Hum. Brain Mapp..

[55] Pawela C.P., Kramer J.M., Hogan Q.H. (2017). Dorsal root ganglion stimulation attenuates the BOLD signal response to noxious sensory input in specific brain regions: Insights into a possible mechanism for analgesia. Neuroimage.

[56] De Ridder D., Plazier M., Kamerling N., Menovsky T., Vanneste S. (2013). Burst spinal cord stimulation for limb and back pain. World Neurosurg..

